# A study on the impact of DRG payment reform on hospitalization costs and outcomes of patients with fractures and intervertebral disc herniations

**DOI:** 10.3389/fpubh.2026.1878859

**Published:** 2026-08-12

**Authors:** Ting Sun, Xinrui Cao, Xuehui Meng, Wenxiu Huang

**Affiliations:** 1 School of Humanities and Management, Zhejiang Chinese Medical University, Hangzhou, China; 2 Department of Health Management Center, Second Affiliated Hospital, School of Medicine, Zhejiang University, Zhejiang, Hangzhou, China

**Keywords:** DRG payment reform, interrupted time series analysis, medical cost control, medical efficiency, traditional Chinese medicine

## Abstract

**Background:**

To curb the growth of medical expenses, the Chinese government has implemented the medical insurance payment reform based on Diagnosis-Related Groups (DRG). This study evaluates the impact of the DRG policy on hospitalization costs, length of stay, and 30-day readmission rates for patients with fractures and intervertebral disc herniation.

**Methods:**

Hospitalization settlement records for patients with fractures and intervertebral disc herniation from a tertiary hospital in a city in eastern China between 2016 and 2025 were selected. An Interrupted Time Series (ITS) analysis was employed, with January 2020 designated as the intervention point.

**Results:**

Following the implementation of the DRG policy, the total hospitalization costs accelerated from a monthly decrease of 0.12% to a monthly decrease of 1.03% (*p* < 0.001). Specifically, costs for the medical insurance group accelerated from a monthly decrease of 0.32 to 0.96% (*p* < 0.01), while the self-pay group shifted from no significant pre-reform trend to a monthly decrease of 1.18% (*p* < 0.001). Western medicine expenses reversed from a pre-reform monthly increase of 0.49% to a monthly decrease of 1.31% (*p* < 0.001). In contrast, Traditional Chinese Medicine (TCM) diagnosis and treatment fees shifted from a monthly decrease of 2.72% to a monthly increase of 1.92% (*p* < 0.01). The rate of decline in length of stay slowed from a monthly reduction of 4.23 to 2.80%, but no statistically significant change was observed (*p* > 0.05). For the medical insurance group, the decrease slowed significantly from 4.83 to 1.61% per month (*p* < 0.01), whereas for the self-pay group, the decrease accelerated from 2.51 to 4.94% per month (*p* > 0.05). No statistically significant change was observed in the 30-day readmission rate before and after the reform (*p* > 0.05).

**Conclusion:**

DRG effectively controlled hospitalization and boosted TCM use without hurting quality. But length of stay kept falling with slower decline, especially in insured patients, showing heterogeneous effects. Policymakers should target payment types to improve equity and effectiveness. Sensitivity and placebo analyses further supported these findings.

## 1 Introduction

Orthopedics, as a key department characterized by concentrated high-value consumables, high surgical volume, and complex cost structures, has long been the focal point for balancing medical insurance cost control with healthcare quality. Conditions such as fractures and intervertebral disc herniation, which exhibit both high incidence rates (1) and significant cost burdens (2, 3), represent key disease categories that account for a substantial portion of medical insurance fund expenditures. Constructing a sustainable medical insurance payment system that safeguards healthcare quality and patient rights while effectively curbing unreasonable cost growth and improving fund utilization efficiency has become a critical issue in the reform of the pharmaceutical and healthcare system (4, 5). Against this backdrop, Diagnosis-Related Groups (DRG) payment—an internationally recognized prepayment method based on case-mix (6)—was introduced to China as a strategic measure for medical insurance payment reform.

The conceptual origins of the DRG system date back to Yale University in the United States during the 1960s. In 1976, the first generation of DRGs (Yale DRGs) was officially established. Its core principle involves grouping cases with similar clinical processes and resource consumption, determining a unified payment standard based on the relative weight of each group, thereby transforming the traditional “fee-for-service” model into “packaged payment by disease type” (7). Subsequently, DRG was widely adopted and developed globally, gradually evolving into several mature versions (8), such as Australia’s AR-DRGs (9, 10), Germany’s G-DRGs (11), and France’s GHS (12). Practical experience from these countries indicates that a successful DRG payment system can not only effectively control costs but also incentivize hospitals to strengthen internal management, shorten average length of stay, and reduce complication rates (13–16). However, international experience has also revealed potential risks associated with DRG (17–23), such as declining service quality due to cost control, “patient dumping” (refusing severe or complicated cases), and “upcoding.” Consequently, countries have continuously iterated their DRG systems through practices such as refining grouping, adjusting weights, and establishing special payment mechanisms (e.g., additional compensation for new technologies or critical/severe cases) to mitigate inherent drawbacks and pursue the dual goals of quality and cost control.

China’s exploration of DRG started relatively late but has progressed rapidly. Chinese scholars began introducing and researching DRG in the early 21st century, with large hospitals and research institutions in cities like Beijing and Shanghai conducting small-scale simulations and data calculations. In 2011, Beijing pioneered DRG payment pilots (BJ-DRG) in six tertiary hospitals, primarily focusing on internal hospital performance management and cost control rather than medical insurance payments. While this phase accumulated valuable experience for national promotion, it did not yet constitute an institutional reform at the national level. In 2019, China incorporated DRG-related indicators into its assessment system, strongly promoting DRG application at the national level. In the same year, the National Healthcare Security Administration (NHSA) announced a list of 30 national pilot cities for DRG payment and released 376 core DRG groups (CHS-DRG). In 2020, pilot cities launched actual payments as scheduled, marking China’s official entry into the era of DRG payment.

Despite significant progress in China’s DRG reform, its actual effects remain under observation and evaluation as a systemic project with profound impacts on the medical ecosystem. Currently, a substantial body of domestic literature explores the impact of DRG payment reform, focusing primarily on total hospitalization costs, average length of stay and readmission rates, and the behavior of hospitals and physicians. Multiple studies indicate that the effects of DRG payment reform vary across costs (24–26), efficiency (24, 27), and quality (27–29), with potential heterogeneity across different regions, hospital levels, and specialties (26, 30, 31). Therefore, high-quality empirical research tailored to specific scenarios is urgently needed for validation and guidance. Moreover, existing studies exhibit several limitations. First, most studies cover short time spans, relying only on 1–3 years of post-reform data, making it difficult to distinguish short-term shocks from long-term adaptation effects or establish reliable baseline trends for comparison. Second, many studies adopt a single perspective, focusing on costs or a single efficiency indicator, and lack a linked analysis of multi-dimensional metrics such as cost structures (e.g., Western medicine costs, Traditional Chinese Medicine (TCM) service fees), patients’ direct economic burden (out-of-pocket ratio), and medical quality and safety (readmission rates). Furthermore, some studies use broad research subjects, often sampling entire hospitals or all inpatients, thereby overlooking the significant differences between departments (e.g., high-cost orthopedics) and disease types, resulting in less targeted conclusions. Finally, given the widespread application of TCM services in the Chinese healthcare context, most studies lack consideration of “TCM service fees,” and related research is particularly scarce, leaving the cost-effectiveness of TCM diagnosis and treatment and its positioning within the DRG framework unclear.

As a pioneer in China’s DRG payment reform, Zhejiang Province has extended DRG-based payment methods to medical institutions across the province since 2020. This province-wide reform covers all inpatient treatment categories and is characterized by broad coverage, large implementation scale, and high data transparency. This study proposes to collect 10 years (2016–2025) of single-center data from a tertiary hospital in Zhejiang Province, constructing a complete observation window spanning 4 years pre-reform and 6 years post-reform. This design effectively controls for confounding factors such as time trends, allowing for a more precise assessment of the net effect of the reform. By focusing on specific high-incidence orthopedic conditions (fractures and disc herniations), the study’s conclusions will possess greater specialist representativeness and clinical guidance value. More importantly, the research attempts to systematically compare changes in six core indicators within a unified framework: total hospitalization costs, cost structure (Western medicine fees, TCM service fees), patient out-of-pocket expenses, average length of stay, and 30-day readmission rates. The aim is to comprehensively depict the “cost-efficiency-quality” landscape triggered by the DRG reform in these patients, with particular attention to the evolution of “TCM service fees”—a characteristic element of Chinese healthcare—to provide a unique perspective for understanding the interaction between DRG reform and TCM.

## 2 Methods

### 2.1 Data source and study population

This study extracted inpatient medical record front sheets and hospitalization settlement data for orthopedic patients admitted to the Second Affiliated Hospital of Zhejiang University School of Medicine between January 2016 and December 2025. All data were anonymized to protect patient privacy. During the data cleaning phase, records with missing entries, irregular admission or discharge durations, and abnormal expenditure values were excluded. The final analytical dataset comprised 33,619 participants.

### 2.2 Outcome measures

This study aimed to examine the impact of the DRG reform on inpatient service outcome indicators among orthopedic patients and to assess differences across subgroups. Accordingly, three dimensions of outcome variables were evaluated: cost-related expenditures (total hospitalization costs, out-of-pocket costs, TCM-related costs, (including diagnosis and treatment fees, Chinese patent medicine fees and Chinese herbal medicine fees) and Western medicine costs), hospital efficiency (length of stay), and quality of care (30-day readmission rate). To account for the effects of inflation and other factors on costs, all average monthly cost were standardized using the annual Chinese Consumer Price Index (CPI) with 2016 as the baseline year, ensuring that the findings reflect real changes in medical expenditures. Given that healthcare cost data are typically skewed, a natural logarithmic transformation was applied to the cost variables for each hospitalized patient. This transformation stabilized variance, facilitated statistical analysis, and more effectively highlighted trends and outliers in medical expenditures. Length of stay was calculated as the difference between the admission and discharge dates. The 30-day readmission rate was calculated for each month, with the total number of hospitalizations in that month as the denominator and the number of patients readmitted within 30 days as the numerator.

### 2.3 Statistical analysis

Before conducting descriptive analyses, propensity score matching (PSM) was performed to balance baseline characteristics between the pre-reform and post-reform groups. A 1:2 nearest-neighbor matching method was adopted, with a caliper value set at 0.2 standard deviations of the propensity score to ensure matching quality. The matching procedure was implemented using the MatchIt package in R 4.4.3, with a random seed set to 123 to ensure reproducibility of the results.

Descriptive analyses were performed using SPSS 25.0. Continuous variables were presented as mean (standard deviation), and categorical variables were expressed as frequency (percentage). To assess the impact of DRG policy implementation on patient characteristics and outcome variables, t-tests and chi-square tests were conducted before and after the reform, taking into account the influence of insurance type.

Furthermore, to evaluate the effects of the DRG policy intervention on patient costs, length of stay, and 30-day readmission rates, an interrupted time series analysis (ITSA) was employed, with January 2020 designated as the intervention point. The general form of the ITSA regression model was specified as follows:

$Y_{t}=\beta_{0}+\beta_{1}\;\text{time}+\beta_{2}\;\text{intervention}+\beta_{3}\;\text{post}+\varepsilon_{t}$ .

In this model, the outcome variable *Y*_t_ represents the outcome at a specific time point t (i.e., the mean of the costs by log-transformed and length of stay for patients admitted that month, as well as 30-day readmission rate monthly). *β*_0_ estimates the baseline level of the outcome variable at the start of the study period. *β*₁ represents the monthly slope of the outcome variable before the DRG policy intervention. *β*₂ denotes the immediate change in the outcome variable at the time of the DRG reform. *β*₃ indicates the change in trend of the outcome variable after the intervention, relative to the trend expected based on the pre-intervention period. *β*₁ + *β*₃ represents the trend immediately following the intervention. The variable “time” represents the time variable, ranging from the beginning to the end of the study period. The variable “intervention” indicates whether the policy intervention had occurred, taking a value of 0 before the intervention and 1 after the intervention. The variable “post” refers to the time series following the intervention, and ε_t_ is the random error term. To address potential autocorrelation in the model, an ordinary least squares (OLS) model was initially fitted using the Newey–West estimator with zero lag. Subsequently, the “actest” command was used to test for autocorrelation. If first-order autocorrelation was detected, the regression was re-estimated using the Prais–Winsten method. For second-order or higher-order autocorrelation, the Newey–West method was applied for adjustment, specifying the necessary number of lags. All tests with a *p*-value < 0.05 were considered statistically significant. All statistical analyses were performed using Stata version 18.0 for Windows.

### 2.4 Ethical approval and informed consent

The study protocol was approved by the Institutional Review Board of the Second Affiliated Hospital of Zhejiang University School of Medicine (Approval No. [2026] Ethics Review No. 0252). All study procedures were conducted in accordance with the ethical guidelines of our institution and the principles of the Declaration of Helsinki. Due to the retrospective nature of the study and the use of anonymized data, the requirement for obtaining individual informed consent was waived by the ethics committee.

## 3 Results

### 3.1 Propensity score matching (PSM)

Before PSM, significant differences were observed between the pre-reform group (n = 8,766) and the post-reform group (n = 24,853) in terms of age (*p* < 0.001), sex (*p* = 0.032), and surgery proportion (*p* < 0.001), indicating the presence of potential confounding bias. After propensity score matching, 8,766 patients in the pre-reform group were successfully matched with 17,221 patients in the post-reform group, yielding a total sample size of 25,987. Following matching, the two groups achieved good balance in age (*p* = 0.357, SMD = 0.012), sex (*p* = 0.664, SMD = 0.006), and surgery proportion (*p* < 0.001, SMD = 0.057), with all covariates having SMD values below 0.1 (Supplementary Table S1). These results indicate that PSM effectively eliminated baseline differences between the two groups, and the matched sample demonstrated good comparability.

### 3.2 Descriptive analysis

Table 1 presents the comparison of baseline characteristics and inpatient service outcome indicators between the pre-reform and post-reform groups. After matching, no statistically significant differences were observed in age or sex between the two groups. The distribution of payment methods changed significantly (*p* < 0.01), with an increase in the proportion of insured patients and a decrease in the proportion of self-paying patients after the reform. Regarding outcome indicators, total hospitalization costs, out-of-pocket costs, TCM-related costs, and Western medicine costs were all significantly lower in the post-reform group compared with the pre-reform group (*p* < 0.01). The average length of stay decreased from 9.07 days before the reform to 6.54 days after the reform (*p* < 0.01). The 30-day readmission rate also decreased from 1.6 to 1.1% (*p* = 0.002).

**Table 1:** Basic characteristics and inpatient outcomes of orthopedic patients before and after the DRG payment reform.

Variables	Before DRG reform					After DRG reform							***p***
	Year				Total	Year						Total	
	2016	2017	2018	2019		2020	2021	2022	2023	2024	2025		
Discharge cases, No.	1911	2025	2,259	2,571	8,766	2,673	3,530	3,600	3,789	2,470	1,159	17,221	
Age, mean (SD)	48.38 (16.98)	49.24 (16.75)	50.13 (16.83)	48.89 (16.75)	49.47 (16.83)	50.00 (16.80)	50.04 (16.86)	49.62 (17.40)	50.50 (16.90)	48.43 (16.20)	48.01 (17.24)	49.68 (16.92)	0.357
Sex, No. (%)													0.654
Male	1,066 (55.8)	1,133 (56)	1,223 (54.1)	1,408 (54.8)	4,830 (55.1)	1,485 (55.6)	1951 (55.3)	2005 (55.7)	2,117 (55.9)	1,365 (55.3)	616 (53.1)	9,539 (55.4)	
Female	845 (44.2)	892 (44)	1,036 (45.9)	1,163 (45.2)	3,936 (44.9)	1,188 (44.4)	1,579 (44.7)	1,595 (44.3)	1,672 (44.1)	1,105 (44.7)	543 (46.9)	7,682 (44.6)	
Medical insurance, No. (%)													<0.01
UEBMI	944 (49.4)	1,064 (52.5)	1,252 (55.4)	1,447 (56.3)	4,707 (53.7)	1,614 (60.4)	1989 (56.3)	1717 (47.7)	2,105 (55.6)	1,076 (43.6)	395 (34.1)	8,896 (51.7)	
URRBMI	34 (1.8)	14 (0.7)	30 (1.3)	23 (0.9)	101 (1.2)	55 (2.1)	106 (3)	357 (9.9)	225 (5.9)	489 (19.8)	339 (29.2)	1,571 (9.1)	
Self-pay	933 (48.8)	947 (46.8)	977 (43.2)	1,101 (42.8)	3,958 (45.2)	1,004 (37.6)	1,435 (40.7)	1,526 (42.4)	1,459 (38.5)	905 (36.6)	425 (36.7)	6,754 (39.2)	
Total hospitalization costs, mean (SD), RMB	30835.53 (23682.94)	28796.48 (20856.67)	30111.20 (24828.51)	28123.06 (22496.74)	29382.29 (23043.83)	24953.44 (19061.12)	26886.08 (20698.79)	27357.47 (24955.45)	22988.90 (21008.33)	20267.90 (32766.45)	15944.56 (16294.29)	24141.55 (23537.10)	<0.01
Out-of-pocket costs, mean (SD), RMB	22187.39 (22126.91)	20793.62 (21002.36)	20283.06 (25486.77)	18548.20 (21100.80)	20307.33 (22542.68)	15299.14 (18572.41)	17537.10 (20446.92)	16155.59 (25475.82)	12801.63 (21738.67)	12861.75 (323813.37)	9949.47 (15591.37)	14677.78 (23547.25)	<0.01
TCM costs, mean (SD), RMB	211.40 (260.89)	349.09 (418.33)	402.25 (443.21)	140.23 (240.89)	271.52 (366.74)	25.60 (137.29)	123.04 (240.48)	148.22 (233.02)	155.64 (243.70)	77.69 (675.14)	136.58 (351.02)	114.76 (338.98)	<0.01
Western medicine costs, mean (SD), RMB	4617.58 (6559.17)	5207.12 (7104.24)	5901.54 (8537.71)	4916.35 (6433.70)	5172.27 (7222.76)	3864.60 (4816.02)	4602.01 (5394.78)	5241.14 (8882.80)	4202.23 (6092.81)	3267.80 (5980.62)	2090.80 (4096.03)	4172.82 (6416.98)	<0.01
Length of stay, mean (SD), day	9.81 (9.96)	9.52 (7.29)	8.87 (6.76)	8.33 (6.66)	9.07 (7.68)	7.19 (6.31)	6.76 (5.98)	6.16 (5.35)	6.55 (5.51)	6.55 (5.52)	5.45 (5.40)	6.54 (5.72)	<0.01
30-day readmission, No. (%)	29 (1.5)	24 (1.2)	37 (1.6)	47 (1.8)	137 (1.6)	25 (0.9)	53 (1.5)	35 (1.0)	42 (1.1)	26 (1.1)	10 (0.9)	191 (1.1)	0.002

### 3.3 Results of interrupted time series analysis (ITSA)

**Figure 1:**
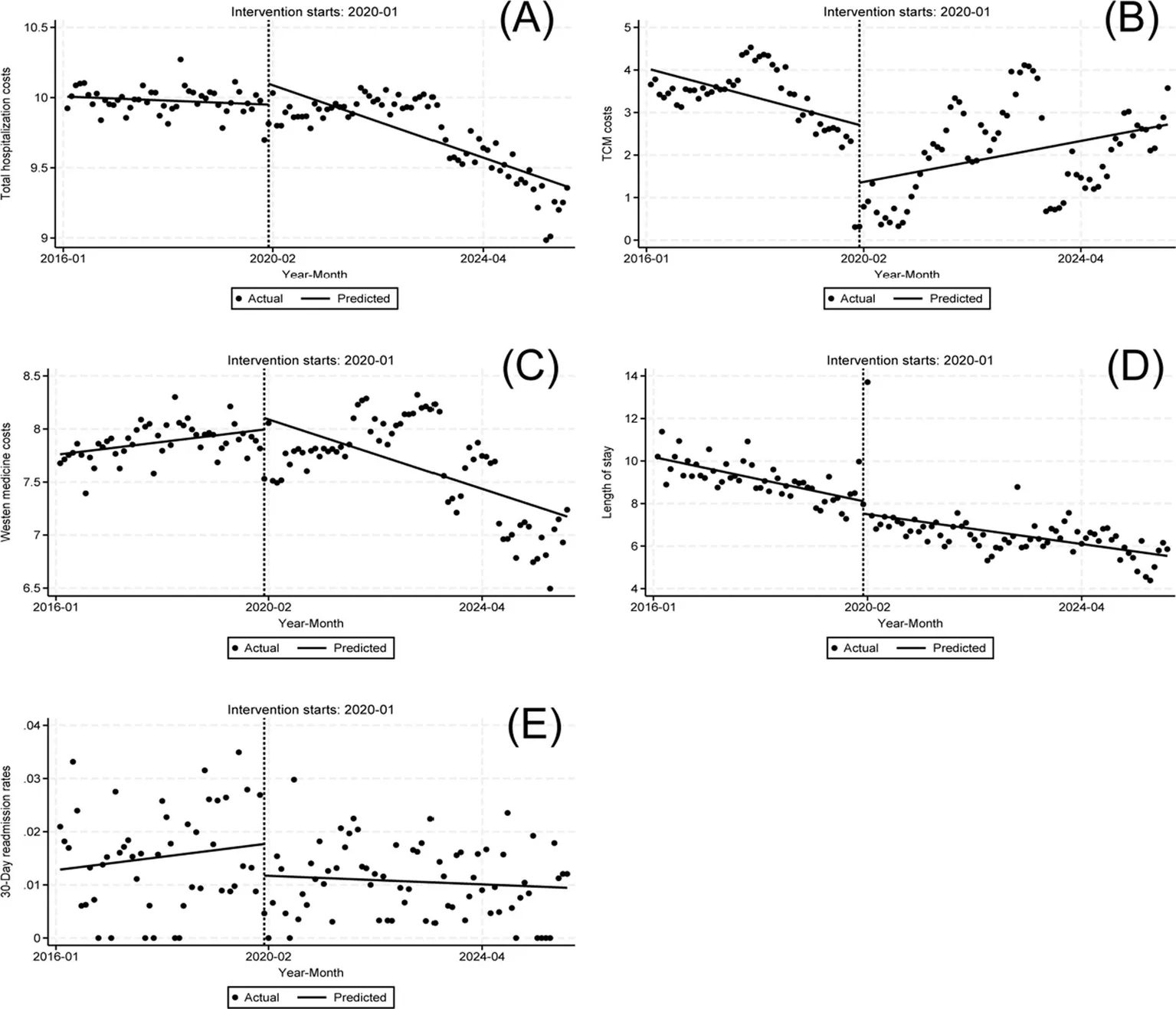
ITSA results of outcomes for inpatient orthopedic patients before and after the DRG payment reform. **(A)** Total hospitalization costs; **(B)** TCM costs; **(C)** Western medicine costs; **(D)** Length of stay; **(E)** 30-day readmission rates.

An interrupted time series model was used to evaluate the impact of the DRG reform on inpatient service outcomes among orthopedic patients, with January 2020 set as the intervention point. Monthly trends before and after the reform were estimated, and the results are shown in Table 2 and Figures 1A–E.

**Table 2:** ITSA results of outcomes for inpatient orthopedic patients before and after the DRG payment reform.

Variable	Baseline monthly slope (β1)	Step change (β2)	Monthly slope change (β3)	Constant (β0)
	Estimate (95%CI)	Estimate (95%CI)	Estimate (95%CI)	Estimate (95%CI)
Total hospitalization costs	−0.0012 (−0.0030, 0.0006)	0.1484 (−0.0204229, 0.3172451)	−0.0091 (−0.013937, −0.0043328)***	10.0069 (9.960587, 10.05311)***
TCM costs	−0.0272 (−0.0496692, −0.0046461)*	−1.3577 (−2.325748, −0.3895952)**	0.0464 (0.017428, 0.0753901)**	4.0102(3.459164, 4.561316)***
Western medicine costs	0.0049 (0.0014391, 0.0082962)***	0.1083 (−0.1894199, 0.4059867)	−0.0180 (−0.0259741, −0.0099269)***	7.7613 (7.67121, 7.851453)***
Length of stay	−0.0423 (−0.0583563, −0.0263358)***	−0.5854 (−1.415548, 0.2446677)	0.0143 (−0.0076698, 0.0363549)	10.1444 (9.73595, 10.55284)***
30-day readmission rates	0.0001 (−0.0000936, 0.0002942)	−0.0059 (−0.0124472, 0.0005491)	−0.0001 (−0.0003432, 0.0000784)	0.0129(0.0076505, 0.018092)***

*p < 0.05, **p < 0.01, ***p < 0.001.

#### 3.3.1 Total hospitalization costs

As shown in Figure 1A, the changes in total hospitalization costs before and after the reform were analyzed. The baseline level of total hospitalization costs was 1000.69% (*β*₀ = 10.0069, *p* < 0.001). Before the reform, total hospitalization costs showed a slight monthly decrease of 0.12%, but this was not statistically significant (*β*₁ = −0.0012, *p* > 0.05). The immediate change in total hospitalization costs following the reform was an increase of 14.84%, but this was not statistically significant (*β*₂ = 0.1484, *p* > 0.05). After the reform, total hospitalization costs decreased by 1.03% per month (*β*₃ = −0.0091, *p* < 0.001), indicating a significant acceleration in the declining trend.

#### 3.3.2 TCM-related costs

Figure 1B presents the changes in TCM-related costs over the study period. The baseline level of TCM-related costs was 401.02% (*β*₀ = 4.0102, *p* < 0.001). Before the reform, TCM-related costs decreased by 2.72% per month (*β*₁ = −0.0272, *p* < 0.05). At the immediate point of reform implementation, there was a significant immediate level change (reduction) of 135.77% (*β*₂ = −1.3577, *p* < 0.01). After the reform, the trend reversed to a monthly increase of 1.93% (*β*₃ = 0.0464, *p* < 0.01), suggesting a deceleration in the declining trend.

#### 3.3.3 Western medicine costs

Figure 1C shows the changes in Western medicine costs before and after the reform. The baseline level of Western medicine costs was 776.13% (*β*₀ = 7.7613, *p* < 0.001). Before the reform, Western medicine costs increased by 0.49% per month (*β*₁ = 0.0049, *p* < 0.001). The immediate change in Western medicine costs following the reform was an increase of 10.83%, but this was not statistically significant (*β*₂ = 0.1083, *p* > 0.05). After the reform, the trend reversed to a monthly decrease of 1.31% (*β*₃ = −0.0180, *p* < 0.001).

#### 3.3.4 Length of stay

Figure 1D illustrates the changes in the length of stay before and after the reform. The baseline level of length of stay was 1014.44% (*β*₀ = 10.1444, *p* < 0.001). Before the reform, length of stay decreased by 4.23% per month (*β*₁ = −0.0423, *p* < 0.001). At the immediate point of reform implementation, there was an immediate level change (reduction) of 58.54%, but this was not statistically significant (*β*₂ = −0.5854, *p* > 0.05). After the reform, the declining trend slowed to a monthly decrease of 2.80% (*β*₃ = 0.0143, *p* > 0.05), although the change in slope was not statistically significant.

#### 3.3.5 30-day readmission rates

Figure 1E displays the trend in the 30-day readmission rate throughout the observation period. The baseline level of 30-day readmission rates was 1.29% (*β*₀ = 0.0129, *p* < 0.001). Before the reform, the monthly change in 30-day readmission rates was not statistically significant (*β*₁ = 0.0001, *p* > 0.05). The immediate change in 30-day readmission rates following the reform was a reduction of 0.59%, but this was not statistically significant (*β*₂ = −0.0059, *p* > 0.05). After the reform, the monthly change remained non-significant (*β*₃ = −0.0001, *p* > 0.05), indicating that the DRG reform did not have a significant impact on readmission rates.

### 3.4 Subgroup analyses

#### 3.4.1 Insured group and self-pay group

**Figure 2:**
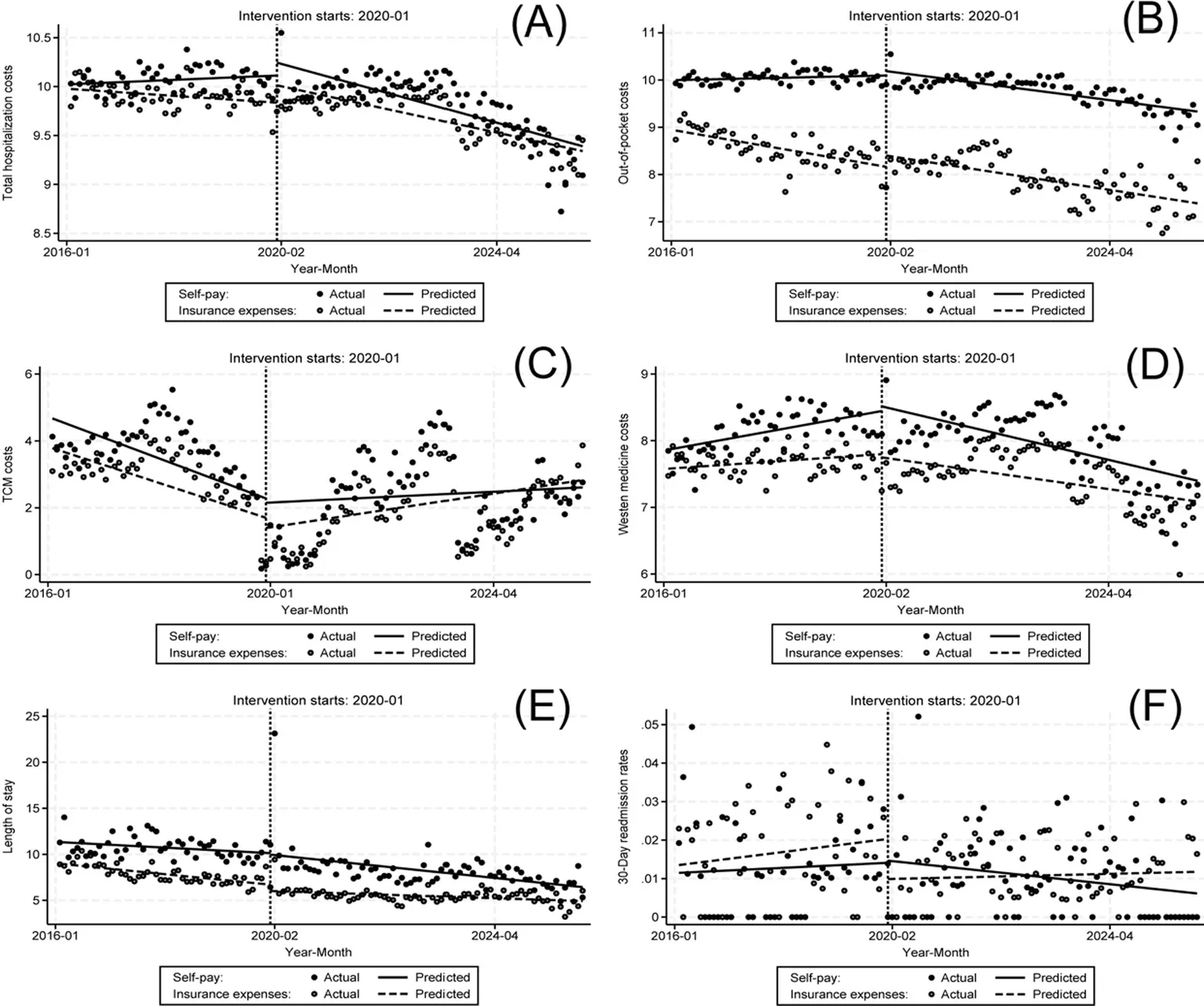
Results of ITSA by payment method groups. **(A)** Total hospitalization costs. **(B)** Out-of-pocket costs. **(C)** TCM costs. **(D)** Western medicine costs. **(E)** Length of stay. **(F)** 30-day readmission rates.

Table 3 and Figures 2A–F present the monthly trends in inpatient service outcomes between the insured group and the self-pay group before and after the DRG reform. The two groups exhibited distinct patterns across multiple indicators.

**Table 3:** Results of ITSA by payment method groups.

Variable	Baseline monthly slope (β1)	Step change (β2)	Monthly slope change (β3)	Constant (β0)
	Estimate (95%CI)	Estimate (95%CI)	Estimate(95%CI)	Estimate (95%CI)
Total hospitalization costs				
Insurance expenses	−0.0032 (−0.0056, −0.0007)*	0.1774 (0.0378, 0.3170)*	−0.0064 (−0.0106, −0.0021)**	9.9866 (9.9146, 10.0586)***
Self-pay	0.0016 (−0.0008, 0.0040)	0.1382 (−0.0685, 0.3450)	−0.0134 (−0.0195, −0.0073)***	10.0270 (9.9658, 10.0881)***
Out-of-pocket costs				
Insurance expenses	−0.0160 (−0.0249, −0.0072)***	0.2391 (−0.0735, 0.5517)	0.0018 (−0.0084, 0.0120)	8.9319 (8.6891, 9.1747)***
Self-pay	0.0018 (−0.0006, 0.0043)	0.0958 (−0.0848, 0.2765)	−0.0138 (−0.0190, −0.0086)***	10.0029 (9.9441, 10.0617)***
TCM costs				
Insurance expenses	−0.0257 (−0.0427, −0.0086)**	−1.3173 (−2.0654, −0.5692)**	0.0480 (0.0253, 0.0707)***	3.6504 (3.2185, 4.0823)***
Self-pay	−0.0263 (−0.0585, 0.0059)	−1.3974 (−2.7854, −0.0093)*	0.0406 (−0.0008, 0.0820)	4.3860 (3.6256 5.1463)***
Western medicine costs				
Insurance expenses	0.0005 (−0.0022, 0.0032)	0.1597 (−0.1544, 0.4739)	−0.0117 (−0.0197, −0.0037)**	7.6500 (7.5664, 7.7335)***
Self-pay	0.0114 (0.0042, 0.0186)**	0.1086 (−0.2361, 0.4533)	−0.0274 (−0.0383, −0.0165)***	7.8758 (7.6834, 8.0683)***
Length of stay				
Insurance expenses	−0.0483 (−0.0678, −0.0288)***	−0.6285 (−1.2368, −0.0203)*	0.0322 (0.0082, 0.0561)**	8.9902 (8.4648, 9.5157)***
Self-pay	−0.0251 (−0.0478, −0.0025)*	−0.1616 (−1.7294, 1.4063)	−0.0243 (−0.0628, 0.0142)	11.3295 (10.6317, 12.0273)***
30-Day readmission rates				
Insurance expenses	0.0001 (−0.0001, 0.0004)	−0.0104 (−0.0188, −0.0020)*	−0.0001 (−0.0004, 0.0001)	0.0135 (0.0073, 0.0198)***
Self-pay	0.0001 (−0.0002, 0.0003)	0.0006 (−0.0079, 0.0090)	−0.0002 (−0.0005, 0.0001)	0.0115 (0.0029, 0.0201)**

*p < 0.05, **p < 0.01, ***p < 0.001.

As shown in Figure 2A, the trends in total hospitalization costs were compared between the two groups. The insured group already showed a monthly decrease of 0.32% before the reform (*β*₁ = −0.0032, *p* < 0.05), and the declining trend accelerated further to 0.96% per month after the reform (*β*₃ = −0.0064, *p* < 0.01). In contrast, the self-pay group showed no significant trend before the reform (*β*₁ = 0.0016, *p* > 0.05) but experienced a significant monthly decrease of 1.18% after the reform (*β*₃ = −0.0134, *p* < 0.001). Both groups demonstrated positive effects of the DRG reform on cost control, with a slightly greater magnitude of reduction observed in the self-pay group.

Figure 2B reveals that the differences in out-of-pocket costs between the two groups were more pronounced. The insured group exhibited a significant monthly decrease of 1.60% before the reform (*β*₁ = −0.0160, *p* < 0.001), but the declining trend markedly slowed after the reform, with no statistical significance (*β*₃ = 0.0018, *p* > 0.05). The self-pay group, however, showed an increasing trend before the reform (*β*₁ = 0.0018, *p* > 0.05) and subsequently transitioned to a significant monthly decrease of 1.20% after the reform (*β*₃ = −0.0138, *p* < 0.001). These findings suggest that the DRG reform effectively reduced the financial burden on patients.

Figure 2C illustrates that both groups showed a rebound trend in TCM-related costs. The insured group experienced a monthly decrease of 2.57% before the reform (*β*₁ = −0.0257, *p* < 0.01), which reversed to a monthly increase of 2.23% after the reform (*β*₃ = 0.0480, *p* < 0.001). The self-pay group also showed a non-significant decreasing trend before the reform (*β*₁ = −0.0263, *p* > 0.05), which turned into an increasing trend after the reform (*β*₃ = 0.0406, *p* > 0.05), although the latter was not statistically significant. These results indicate that the DRG reform further promoted the application and development of TCM.

As shown in Figure 2D, both groups achieved effective cost control in Western medicine costs after the reform. The insured group showed a monthly decrease of 1.12% after the reform (*β*₃ = −0.0117, *p* < 0.01). The self-pay group reversed from a monthly increase of 1.14% before the reform (*β*₁ = 0.0114, *p* < 0.01) to a monthly decrease of 1.60% after the reform (*β*₃ = −0.0274, *p* < 0.001).

Figure 2E shows that both groups exhibited declining trends in the length of stay. The insured group showed a monthly decrease of 4.83% before the reform (*β*₁ = −0.0483, *p* < 0.001), with a significant deceleration in the declining trend after the reform (*β*₃ = 0.0322, *p* < 0.01). The self-pay group experienced a monthly decrease of 2.51% before the reform (*β*₁ = −0.0251, *p* < 0.05), and the declining trend further accelerated to 4.94% per month after the reform, although the change in slope was not statistically significant (*β*₃ = −0.0243, *p* > 0.05).

As shown in Figure 2F, no significant changes in 30-day readmission rates were observed before or after the reform in either group, suggesting that the DRG reform did not adversely affect patients’ short-term readmission risk.

#### 3.4.2 Employee insurance group and resident insurance group

**Figure 3:**
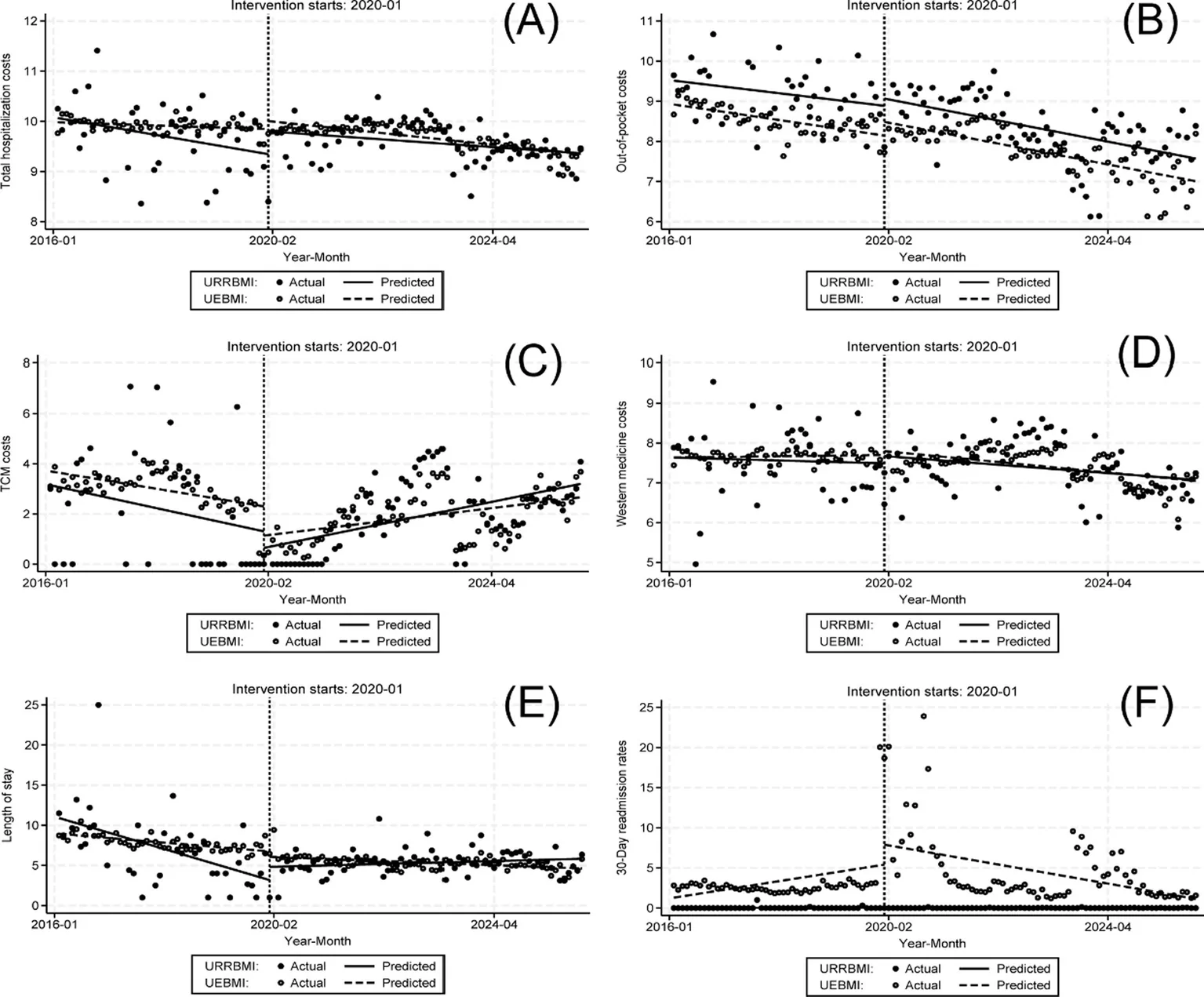
Results of ITSA by health insurance category: **(A)** Total hospitalization costs, **(B)** Out-of-pocket costs, **(C)** TCM costs, **(D)** Western medicine costs, **(E)** Length of stay, and **(F)** 30-day readmission rates. UEBMI, Employee health insurance; URRBMI, Resident health insurance.

We additionally performed subgroup analyses by health insurance type (employee vs. resident insurance); the specific results are shown in Table 4 and Figures 3A–F. Figures 3A and 3D reveal that, with respect to cost control, the employee insurance group exhibited a more pronounced decreasing trend in total hospitalization costs and Western medicine costs. In terms of alleviating patients’ direct financial burden, the resident insurance group showed a more substantial reduction in out-of-pocket costs (see Figure 3B). Figure 3C shows that TCM-related costs rebounded in both groups, with a greater increase observed in the resident insurance group. Figure 3E reveals that both groups experienced a decline in the length of stay; however, the rate of decline slowed in the employee insurance group while accelerating in the resident insurance group. As shown in Figure 3F, the 30-day readmission rate remained stable in the resident insurance group but showed some fluctuations in the employee insurance group.

**Table 4:** Results of ITSA by health insurance category.

Variable	Baseline monthly slope (β1)	Step change (β2)	Monthly slope change (β3)	Constant(β0)
	Estimate (95%CI)	Estimate (95%CI)	Estimate (95%CI)	Estimate (95%CI)
Total hospitalization costs				
UEBMI	−0.0029 (−0.0053, −0.0004)*	0.1598 (0.02839, 0.2913)*	−0.0063 (−0.0106, −0.0021)**	9.9821 (9.9110, 10.0532)***
URRBMI	−0.0153 (−0.0277, −0.0029)*	0.4683 (0.0709, 0.8656)*	0.0089 (−0.0044, 0.0223)	10.0771 (9.6751, 10.4791)***
Out-of-pocket costs				
UEBMI	−0.0164 (−0.0221, −0.0108)***	0.3588 (0.1005, 0.6171)**	−0.0047 (−0.0133, 0.0039)	8.9262 (8.7837, 9.0686)***
URRBMI	−0.0142 (−0.0277, −0.0006)*	0.2231 (−0.2364, 0.6827)	−0.0075 (−0.0228, 0.0077)	9.5490 (9.1401, 9.9579)***
TCM costs				
UEBMI	−0.0257 (−0.0422, −0.0093)**	−1.3663 (−2.0847, −0.6480)***	0.0482 (0.0259, 0.0704)***	3.6681 (3.2513, 4.0849)***
URRBMI	−0.0398 (−0.0959, 0.0163)	−0.67671 (−2.3576, 1.0042)	0.0748 (0.0160, 0.1335)*	3.2599 (1.5853, 4.9344)***
Western medicine costs				
UEBMI	0.0005 (−0.0022, 0.0031)	0.1350 (−0.1640, 0.4341)	−0.0114 (−0.0191, −0.0036)**	7.6519 (7.5676, 7.7361)***
URRBMI	−0.0024 (−0.0250, 0.0202)	0.2549 (−0.3627, 0.8726)	−0.0078 (−0.0320, 0.0164)	7.6149 (6.8677, 8.3620)***
Length of stay				
UEBMI	−0.0459 (−0.0653, −0.0264)***	−0.6543 (−1.2596, −0.0489)*	0.0252 (0.0016, 0.0489)*	8.9410 (8.4224, 9.4597)***
URRBMI	−0.1619 (−0.2477, −0.0762)***	1.7060 (−0.2505, 3.6625)	0.1752 (0.0875, 0.2630)***	10.9284 (7.9502, 13.9067)***
30-Day readmission rates				
UEBMI	0.0471 (−0.0345, 0.1287)	4.6068 (1.0242, 8.1893)*	−0.1592 (−0.2815, −0.0369)*	1.8325 (0.4694, 3.1956)**
URRBMI	0.0004 (−0.0014, 0.0021)	−0.0342 (−0.0869, 0.0184)	−0.0002 (−0.0020, 0.0015)	0.0230 (−0.0436, 0.0895)

*p < 0.05, **p < 0.01, **p < 0.001.

UEBMI, employee health insurance; URRBMI, resident health insurance.

#### 3.4.3 Disc herniation group and fracture group

Subgroup analyses by disease type (disc herniation vs. fracture) were also performed, and the results are shown in Supplementary Table S2 and Supplementary Figures S1A–F. Supplementary Figures S1A,B,D reveal that both groups exhibited decreasing trends in total hospitalization costs, out-of-pocket costs, and Western medicine costs after the reform, with the fracture group showing a greater magnitude of cost reduction. Supplementary Figure S1C illustrates that TCM-related costs rebounded in both groups, with a more significant increase observed in the disc herniation group. Regarding the length of stay, Supplementary Figure S1E shows that the fracture group maintained a sustained decline after the reform, whereas the decreasing trend slowed in the disc herniation group. As shown in Supplementary Figure S1F, the 30-day readmission rate did not change significantly in either group, suggesting that the DRG reform did not adversely affect the short-term readmission risk for patients with either disease condition.

### 3.5 Robustness test

To mitigate potential confounding from the COVID-19 pandemic, which coincided with the DRG implementation in January 2020, we conducted a robustness check by excluding the 2019–2022 period and comparing outcomes between the pre-pandemic period (2016–2018) and the post-pandemic period (2023–2025). Results showed that total hospitalization costs and length of stay declined after the reform, while the 30-day readmission rate remained stable. These findings are directionally consistent with the main ITSA results, supporting the robustness of our conclusions.

To further validate that the observed decline in total hospitalization costs was attributable to the DRG policy intervention rather than to underlying secular trends, we conducted a placebo test with a falsified intervention date of January 2018. The results show that the slope of total hospitalization costs did not change significantly after the reform (*β*₃ = −0.0013, 95% CI: −0.0060 to 0.0034, *p* > 0.05), which confirms the robustness of our main findings.

## 4 Discussion

This study set January 2020 as the policy intervention point, evaluating the monthly trends in medical costs, efficiency, and quality for the 4 years prior to and 6 years following the implementation of DRG reform. The DRG reform achieved significant results in controlling treatment costs, a finding consistent with previous research conclusions (6, 30). When analyzing the total population—including both self-paying and medical insurance patients—the results showed that the immediate change in total hospitalization costs following the reform was an increase of 14.84%, which was not statistically significant. On the one hand, this may be attributed to the fact that clinicians were unfamiliar with DRG grouping rules in the early stage of reform, continued pre-reform clinical practice patterns, and had not yet developed cost control awareness, which offset the immediate cost reduction effect of the reform. On the other hand, as a single-center study, the sample size is often limited and the statistical power is relatively low; monthly or small-sample data are more susceptible to random noise interference, which may lead to insignificant short-term fluctuations in the ITS analysis. Also, without aligned incentives, hospitals may not immediately curb costs, which may also lead to an immediate increase in total hospitalization costs. The rate of monthly decline in total hospitalization costs accelerated significantly after the reform. Subgroup analysis revealed that the medical insurance group already exhibited a pre-reform downward trend of 0.32% per month, likely reflecting the incremental nature of medical insurance payment reform. Prior to the full implementation of DRG, medical insurance departments had already managed fund expenditures through tools such as global budget caps. Consequently, medical institutions had accumulated experience in managing costs for insured patients. Post-reform, the rate of decline accelerated further to 0.96% per month. This essentially reflects how the “precision payment” mechanism of DRG strengthened cost-control constraints while guiding hospitals to reduce costs through standardized clinical pathways, regional mutual recognition of examination and test results, and whole-process consumables supervision. The self-paying group showed no significant downward trend before the reform, but experienced a significant post-reform decline of 1.18% per month—exceeding even the rate of the medical insurance group. This result reflects the reshaping effect of the DRG reform on the overall clinical behavior of medical institutions. Once hospitals established refined operational systems to adapt to DRG rules, cost-control measures extended to all patients, not just those covered by insurance.

Post-reform, hospitalization costs decreased for both employees insured by the basic medical insurance for urban employees (employee medical insurance) and residents insured by the basic medical insurance for urban residents (resident medical insurance). However, the decline was faster for the employee medical insurance group, consistent with previous findings (32). This phenomenon may be related to both hospital and patient choices. The higher reimbursement ratio for employee medical insurance means that the insurance-covered portion of the payment for the same DRG group is higher. If a hospital can reduce costs through optimized treatment, the surplus profit obtained from employee medical insurance cases is more substantial. Therefore, hospitals prioritize optimizing clinical pathways and reducing unnecessary drug and consumable use for employee medical insurance patients. Furthermore, as most employee medical insurance enrollees are employed, they possess stronger payment capabilities and tend to choose tertiary hospitals. After the DRG reform, tertiary hospitals, aiming to control costs, actively optimized the diagnosis and treatment processes for high-weight DRG groups—for instance, reducing the use of high-value consumables and shortening the average length of stay. Such adjustments have a more pronounced effect on the average cost per case for employee medical insurance patients. In contrast, resident medical insurance enrollees pay lower premiums and more frequently attend primary medical institutions, where mild cases and common diseases predominate. Their medical costs are inherently lower than those of employee medical insurance patients (33), and the potential for cost reduction is relatively limited.

Of course, a comprehensive and targeted evaluation of cost-control outcomes is crucial. Ultimately, cost control should not be viewed as the ultimate goal of the reform. Instead, the DRG payment system should serve as a management tool and policy lever to guide rational resource allocation and promote value-oriented, precision medical management.

Regarding out-of-pocket expenses, the medical insurance group showed a significant pre-reform decline of 1.60% per month, but the trend slowed post-reform. This trend may be attributed to the treatment of relatively more severe conditions after the reform, as well as provider behaviors such as recommending non-reimbursable drugs or services to compensate for lost income. Conversely, the self-paying group showed an upward trend before the reform, potentially linked to the implicit “fee-for-service” shifts in past public hospital reforms. After the reform, the self-paying group shifted to a significant decline of 1.2% per month. The core reason is that DRG reform extended cost control to all patient hospital-wide. To control the overall cost of the same DRG group, hospitals prioritized the use of volume-based procurement (VBP) drugs, standardized examination and test items, and shortened the average length of stay to reduce fixed costs such as bed and nursing fees for all patients, including self-payers. Simultaneously, policies in many regions explicitly require controlling the proportion of out-of-pocket expenses for inpatients. These results suggest that DRG reform alleviated the burden of out-of-pocket costs for residents, breaking the previous dilemma of “medical insurance patients benefiting more, self-paying patients benefiting less” seen in past reforms.

In terms of Western medicine costs, the immediate change was an increase of 10.83%, which was not statistically significant. This may be related to the initial reduction in the use of high-value consumables and diagnostic tests by hospitals after the DRG reform, where some clinicians substituted postoperative adjuvant therapies and complication prevention regimens with Western medicines of confirmed efficacy, driving up Western medicine costs. Prior to the reform, Western medicine costs increased by 0.49% per month. Following the reform, the trend shifted to a monthly decrease of 1.31%. Both the medical insurance and self-paying groups achieved effective control of Western medicine costs post-reform. DRG reform shifts the traditional “fee-for-service” model to a “case-mix prepayment” system. Under this mechanism, hospitals do not restrict cost control to insured patients but extend pharmaceutical cost management to all inpatients. On one hand, hospitals prioritize cost-effective drugs with equivalent efficacy. On the other hand, they standardize clinical pathways uniformly to reduce the irrational use of adjuvant drugs and antimicrobials. Consequently, hospitals have gradually moved away from a drug-centric revenue model, leading to a more balanced structure of medical expenditures. Although the self-paying group is not directly involved in DRG insurance settlements, they remain subject to the dual constraints of hospital cost-control strategies and policy requirements, driving down Western medicine costs.

The immediate change in TCM-related costs was a decrease of 135.77%, which was statistically significant. This could be explained by the absence of supporting payment policies for TCM services (such as DRG grouping preference for TCM advantageous disease categories, separate payment for TCM services, etc.) in the first month of reform implementation. To rapidly adapt to the “self-payment for overspending, retention of savings” rule, hospitals directly suspended most TCM services (such as canceling TCM rehabilitation programs and discontinuing the prescription of Chinese herbal pieces), leading to the decline in TCM-related costs. Analyzing the total population revealed that the pre-reform monthly decline in TCM-related costs slowed post-reform, shifting to a monthly increase of 1.92%. Subgroup analysis showed an upward trend in TCM-related costs for both the medical insurance and self-paying groups, with a significant increase observed in the employee medical insurance group post-reform. This suggests that DRG reform has further promoted the application and development of TCM. DRG reform is not merely a cost-control tool; in recent years, national and local levels have simultaneously introduced a series of supporting policies to “support TCM development through medical insurance,” providing space for the realization of the value of TCM services through grouping rules, payment standards, and incentive coefficients, directly driving the reasonable recovery of TCM-related costs. On one hand, many regions have innovated DRG grouping logic for TCM preferred disease type, breaking the traditional Western-medicine-focused grouping that overlooked TCM characteristics. Nanjing took the lead nationwide in constructing the NJ-DRG grouper integrating Chinese and Western medicine characteristics, adding 51 characteristic TCM DRG groups and setting higher benchmark points for TCM preferred disease type. On the other hand, regions such as Anhui and Jiangsu have established TCM preferential payment mechanisms. These policies collectively increased the scale of medical insurance payments for TCM services and patient acceptance, driving the overall recovery of TCM-related costs. After the DRG reform, the potential for cost reduction per case for employee medical insurance patients was greater, allowing hospitals more surplus funds to invest in supplying TCM characteristic services. Simultaneously, the employee group has a higher demand for high-quality, personalized medical services and greater acceptance of TCM characteristic services such as acupuncture, tuina, and syndrome differentiation and treatment, being willing to pay higher fees for the value of TCM technical labor, which drives faster growth of TCM-related costs among this group. From the service scenario perspective, employee medical insurance patients are concentrated more in tertiary hospitals, which are hubs for TCM preferred disease type, leading to a higher frequency of TCM service usage among this group, thereby driving the overall increase in diagnosis and treatment fees. In addition, under the DRG bundled payment system, hospitals face a revenue ceiling for Western-medicine DRG groups, and the rule of “overspending self-paid, retaining surplus” mandates cost control. Because TCM services are generally not assigned relative weights or are separately billable, hospitals have a strong financial incentive to strategically expand chargeable TCM items—such as acupuncture, tuina (Chinese medical massage), and Chinese herbal preparations—as marginal revenue sources, and may proactively market them to offset losses from Western-medicine services. A recent Chinese DRG reform study also showed that DRG implementation reduced stroke inpatients’ hospitalization costs and increased the use of TCM treatments in this patient group (34). Furthermore, self-paying patients’ own financial capacity and treatment preferences can also drive the utilization rate of TCM services, thereby contributing to the rebound in TCM-related costs. Price-sensitive populations are more inclined to choose TCM when their out-of-pocket proportion decreases and total costs are lower, collectively leading to an increase in the utilization rate of TCM services.

In terms of medical efficiency, for the total population, the average length of stay decreased by 4.23% per month before the reform. The immediate change in length of stay was a decrease of 58.54%, which was not statistically significant. This may be because hospitals had launched clinical pathway optimization in the first month of reform, planning to implement models such as “day surgery” and “early postoperative discharge,” showing an initial trend of length of stay reduction. However, the large sample fluctuation led to the failure to pass the significance test. And post-reform, the rate of decline slowed slightly to 2.80% per month, but the change was not statistically significant, which may be related to the increased use of TCM treatments. Subgroup analysis showed that for the medical insurance group, the length of stay decreased by 4.83% per month before the reform, slowing significantly post-reform. The pre-reform reduction rate for the medical insurance group was much higher than the total population average, likely directly related to the long-term groundwork of medical insurance payment reform. Before the reform, medical insurance departments had already controlled the length of stay for insured patients through global budgets and other tools, and medical institutions had optimized processes for these patients sufficiently, significantly squeezing the potential for further compression of hospital stays. The significant slowing of the decline post-reform indicates that the length of stay for insured patients had approached a reasonable range, with limited room for further shortening. This highlights the necessity of continuous supervision and monitoring to mitigate policy fatigue and maintain the long-term effectiveness of the reform. Ongoing monitoring, regular adjustment of regulatory tools, and the introduction of supplementary policy measures will be essential to ensure lasting improvements in medical efficiency and cost control under the DRG reform. For the self-paying group, the pre-reform monthly reduction in length of stay was only 2.51%—much lower than the medical insurance group—directly related to the lack of cost-control constraints for self-pay patients in the past. The post-reform acceleration to 4.94% essentially reflects the spillover effect of the hospital-wide operational efficiency improvements driven by DRG reform. Policymakers should target payment types to improve equity and effectiveness. On the one hand, set reasonable length-of-stay benchmarks with incentives for insured patients. Given that insured patients’ length of stay declined more slowly under DRG, policymakers could establish evidence-based length of stay benchmarks for common DRG groups among insured populations and introduce reward mechanisms (e.g., tiered surplus retention) for hospitals that meet them, while using the “special case review” channel to avoid rigid 15-day discharges that harm complex cases. On the other hand, strengthen quality supervision of TCM services for self-paying patients. Since self-pay patients showed faster length of stay reduction yet may drive TCM utilization, specific quality monitoring (e.g., TCM appropriateness evaluation, adverse event tracking) should target self-pay TCM services to prevent overuse masked by shorter stays.

Regarding medical quality, the immediate change in the 30-day readmission rate was a decrease of 0.59%, which was not statistically significant. The impact of DRG on medical quality (such as excessive cost control leading to disease recurrence and increased readmission rates after discharge) usually takes 3 to 6 months to manifest. In the first month of reform, clinicians still maintained cautious diagnosis and treatment for critically ill patients, and did not deliberately shorten the length of stay of critically ill patients for cost control purposes. Therefore, the readmission rate did not fluctuate significantly and only showed a slight decrease. Also, there was no statistically significant monthly change in the 30-day readmission rate for the total population, medical insurance patients, or self-paying patients before or after the reform. This suggests that the DRG reform did not significantly impact readmission rates, implying that clinical outcomes were not sacrificed. Overall, the decrease in total hospitalization costs, the reduction in length of stay, and the stable 30-day readmission rate embody the core goal of “improving quality and efficiency” of the DRG reform. This reform has achieved initial success in promoting a value-based medical model (35).

This study has its strengths. First, the research data covers all patients with fractures and intervertebral disc herniations in the hospital from January 2016 to December 2025, ensuring the completeness of the evaluation data. Second, the study established a control group, conducted analyses across different subgroups, and analyzed multi-dimensional medical indicators under the DRG reform, yielding relatively comprehensive results. However, this study also has limitations. First, this study is based solely on the analysis of selected disease categories from a single provincial-level tertiary general hospital. Constrained by the hospital tier characteristics of the single-center sample and the scope of disease coverage, the generalizability of the research conclusions is subject to certain limitations. While this design choice facilitates the control of confounding factors within a single institution and enhances internal validity, it may render the results difficult to directly extrapolate to broader healthcare settings. The diagnostic capability, resource allocation, and patient disease complexity of provincial tertiary hospitals are significantly higher than those of primary healthcare institutions, and the effects of DRG payment reform vary markedly across hospitals of different tiers. This study used single-center data, and in certain months the sample size was too small, resulting in insufficient statistical power and representing a specific limitation. The study was not seasonally adjusted and no additional quality indicators (e.g., in-hospital infection rates or postoperative complications) were included due to data limitations. Future research with richer clinical data should further explore these dimensions to provide more comprehensive evidence on quality preservation. Also, this study only analyzed two common orthopedic diseases, failing to comprehensively evaluate the overall impact of DRG reform. To verify the universality of the conclusions, future research could incorporate multicenter data from medical institutions of different tiers and regions to compare the heterogeneity of DRG reform effects. Future studies should extend the analysis to other diseases that rely heavily on high-value consumables (e.g., joint replacement or cardiovascular intervention) to examine the generalizability of the DRG reform effects across different specialties. Although this study identified an association between the DRG reform and the reduction in hospitalization costs via ITSA, it must be noted that the applicability boundary of our conclusion is constrained by certain policy context. The national centralized VBP policy for drugs and surgical consumables may have a superimposed impact on changes in hospitalization costs, and this study did not fully isolate the independent effect of this factor. Future research could further incorporate variables such as the timeline of VBP policy implementation and the scope of covered product categories, to quantitatively disentangle the independent and synergistic effects of DRG reform and VBP policy, so as to more accurately assess the actual impact of individual healthcare reform policies.

## 5 Conclusion

The DRG reform has demonstrated significant efficacy in controlling inpatient and Western medication costs, regardless of patients’ insurance status (i.e., insured or self-paying). This indicates a robust cost-containment effect of the DRG reform, which has reshaped the overall clinical practice of medical institutions. The cost-control measures extend to all patients, rather than targeting insured patients exclusively. Regarding out-of-pocket expenses, the reform facilitated a significant reduction for self-paying patients, suggesting that DRG implementation alleviates the financial burden on residents. Furthermore, TCM-related costs reversed their trend and exhibited a monthly increase post-reform, indicating that DRG reform has promoted the utilization of TCM therapies among patients with fractures and intervertebral disc herniation. Although the length of stay continued to decline post-reform, the rate of decrease decelerated. Specifically, the decline in length of stay for insured patients slowed markedly, while self-paying patients maintained a steady rate of decrease. This may be attributed to the fact that process optimization for insured patients has reached a plateau, leaving limited room for further compression of length of stay. Notably, the 30-day readmission rate showed no statistically significant change, suggesting that the DRG reform did not compromise clinical outcomes.

Overall, the DRG reform effectively curbed medical costs and reduced the financial burden on patients while significantly improving operational efficiency for certain patient groups. As the reform progresses, policymakers should prioritize healthcare equity across different payment types and seek optimal solutions to ensure high-quality medical services for all patients.

## Data Availability

The raw data supporting the conclusions of this article will be made available by the authors, without undue reservation.
