# Supplementary material for: A study on the impact of DRG payment reform on hospitalization costs and outcomes of patients with fractures and intervertebral disc herniations
Source: Front Public Health. 2026 Aug 12;14:1878859. doi: 10.3389/fpubh.2026.1878859 (PMC13507677; doi:10.3389/fpubh.2026.1878859)
Supplement: Supplementary file 1 [file Table_1.docx]

**Table S1 Results of PSM**

| **Variable** | **Before PSM** | | | | | **After PSM** | | | | |
| --- | --- | --- | --- | --- | --- | --- | --- | --- | --- | --- |
|  | **Total (n = 33619)** | **Before reform (n = 8766)** | **After reform (n = 24853)** | ***P*** | **SMD** | **Total (n = 25987)** | **Before reform (n = 8766)** | **After reform (n = 17221)** | ***P*** | **SMD** |
| **Sex, n (%)** |  |  |  |  |  |  |  |  |  |  |
| Male | 18192 (54.1) | 4830 (55.1) | 13362 (53.8) | 0.032 | 0.027 | 14369 (55.3) | 4830 (55.1) | 9539 (55.4) | 0.664 | 0.006 |
| Female | 15427 (45.9) | 3936 (44.9) | 11491 (46.2) |  |  | 11618 (44.7) | 3936 (44.9) | 7682 (44.6) |  |  |
| **Surgery, n (%)** |  |  |  |  |  |  |  |  |  |  |
| 0 | 2246 (6.7) | 848 (9.7) | 1398 (5.6) | <0.001 | 0.153 | 2233 (8.6) | 848 (9.7) | 1385 (8.0) | <0.001 | 0.057 |
| 1 | 31373 (93.3) | 7918 (90.3) | 23455 (94.4) |  |  | 23754 (91.4) | 7918 (90.3) | 15836 (92.0) |  |  |
| **Age, mean (SD)** | 50.19 (17.65) | 49.47 (16.83) | 50.45 (17.92) | <0.001 | 0.056 | 49.61 (16.89) | 49.47 (16.83) | 49.68 (16.92) | 0.357 | 0.012 |

**Table S2 Results of ITSA by Disease Category**

|  | **Baseline monthly slope(β1)** | **Step change(β2)** | **Monthly slope change(β3)** | **Constant(β0)** |
| --- | --- | --- | --- | --- |
|  | **Estimate(95%CI)** | **Estimate(95%CI)** | **Estimate(95%CI)** | **Estimate(95%CI)** |
| **Total hospitalization costs** |  |  |  |  |
| Disc herniation group | -0.0022 (-0.0056, 0.0013) | 0.1492 (0.0057, 0.2927) * | -0.0071 (-0.0122, -0.0021) ** | 9.8812 (9.7952, 9.9672) *** |
| Fracture group | -0.0004 (-0.0031, 0.0024) | 0.1331 (-0.0321, 0.2983) | -0.0110 (-0.0163, -0.0056) *** | 10.0734 (9.9858, 10.1610) *** |
| **Out-of-pocket costs** |  |  |  |  |
| Disc herniation group | -0.0208 (-0.0249, -0.0167) *** | 0.0729 (-0.2368, 0.3826) | 0.0034 (-0.0059, 0.0127) | 9.4122 (9.2969, 9.5274) *** |
| Fracture group | -0.0044 (-0.0097, 0.0008) | 0.1576 (-0.1327, 0.4480) | -0.0102 (-0.0181, -0.0024) * | 9.5015 (9.3511, 9.6520) *** |
| **TCM costs** |  |  |  |  |
| Disc herniation group | -0.0052 (-0.0198, 0.0095) | -1.2721 (-1.7775, -0.7667) *** | 0.0364 (0.0175, 0.0552) *** | 1.8838 (1.4556, 2.3120) *** |
| Fracture group | -0.0361 (-0.0714, -0.0009) * | -1.6536 (-3.1894, -0.1171) * | 0.0501 (0.0040, 0.0963) * | 5.1524 (4.3989, 5.9059) *** |
| **Westen medicine costs** |  |  |  |  |
| Disc herniation group | 0.0013 (-0.0014, 0.0040) | 0.0206 (-0.3041, 0.3452) | -0.0108 (-0.0181, -0.0034) ** | 7.2327 (7.1538, 7.3116) *** |
| Fracture group | 0.0077 (0.0017, 0.0137) * | 0.1364 (-0.1536, 0.4264) | -0.0251 (-0.0350, -0.0152) *** | 8.0587 (7.8910, 8.2263) *** |
| **Length of stay** |  |  |  |  |
| Disc herniation group | -0.0567 (-0.0750, -0.0383) *** | -0.6658 (-1.2958, -0.0358) * | 0.0507 (0.0302, 0.0712) *** | 7.8623 (7.4484, 8.2763) *** |
| Fracture group | -0.0305 (-0.0479, -0.0131) ** | -0.6887 (-1.4959, 0.1185) | -0.0160 (-0.0389, 0.0069) | 11.3897 (10.8644, 11.9151) *** |
| **30-Day readmission rates** |  |  |  |  |
| Disc herniation group | 0.0002 (-0.0002, 0.0006) | -0.0164 (-0.0297, -0.0031) * | -0.0002 (-0.0007, 0.0002) | 0.0202 (0.0108, 0.0296) *** |
| Fracture group | 0.0001 (-0.0001, 0.0002) | -0.0014 (-0.0066, 0.0038) | -0.0001 (-0.0003, 0.0001) | 0.0084 (0.0031, 0.0138) *** |

**Note:** * *P* <0.05, ** *P* <0.01, *** *P* <0.001
